# Harvesting of flow current through implanted hydrophobic PTFE surface within silicone-pipe as liquid nanogenerator

**DOI:** 10.1038/s41598-022-07614-5

**Published:** 2022-03-08

**Authors:** Ravi Kumar Cheedarala, Jung Il Song

**Affiliations:** Department of Mechanical Engineering, Research Institute of Mechatronics, Changwon National University, Changwon City, Republic of Korea

**Keywords:** Energy science and technology, Nanoscience and technology

## Abstract

Harvesting of flow current through implanted hydrophobic surface within silicone pipe as liquid nanogenerators where Tap water (TW), and DI water (DIw) as liquid reservoirs to successfully convert induced mechanical energy into electrical energy. Here, we used a commercial PTFE film for the generation of a hydrophobic surface as a source of mechanical energy. The surface roughness of the hydrophobic surface is confirmed using atomic force microscopy, and contact angle analyses. The generation of power through the interaction of TW and DI with inbuilt PTFE in silicone tube is described. The higher output voltage (V*oc*), and short circuit currents (I*sc*) were attained through an interaction of TW and DIw with N-PTFE. The lower V*oc*, and I*sc*’s were produced when DI water interacts with N-PTFE electrode, whereas TW produced higher V_oc_ and I_sc’s_, respectively, due to a lack of free mobile ions in DIw than TW. The TW-Sh-TENG and DIw-Sh-TENG are produced the maximum peak-to-peak Voc, and Isc of 29.5 V and 17.4 V and 3.7 μA, and 2.9 μA, respectively. Significant power output enhancement of ~ 300% from TW-Sh-TENG from DIw-N-TENG due to the formation of higher surface roughness and lead to the slipping of water droplets by super-hydrophobicity.

## Introduction

Triboelectric nanogenerators (TENGs) can harvest energy from different sources in the environment, including mechanical vibration, heat, tides, and wind^1–6^. Also known as triboelectrification, contact electrification involves surface charge transfer when materials with two different polarities are brought into contact. Various triboelectric mechanisms have been developed using tidal and wind energy to generate mechanical vibration, as well as human movement, muscle stretching, and air and water flow^7–10^. There are many potential applications, such as energy harvesting, chemical sensors, electrostatic-charge patterning, metal-ion detection, and laser printing^11–13^.

A solid TENG is based on solid–solid contact electrification in dry weather, but the efficiency is gradually ruined due to the increasing effect of friction, which damages the effective-contact surface area and decreases the triboelectric charge output due to nano- to micrometer patches between the two solid electrodes^14,15^. Besides, solid–solid contact TENGs become saturated because of the lack of accessibility of surface destruction that could occur with flexible materials^2,16^. It is difficult to examine the yielding power at the nano-level, which defines the point where the material structure begins to change (elastic + plastic) nonlinearly. Therefore, there are currently many technologists focusing on the development of solid–liquid contact TENGs for generating high triboelectricity. But the development of an effective solution for this problem remains a challenging task, and the approaches are expensive and complex^17,18^.

Lin et al. reported a triboelectric charge with an open-circuit voltage (V_*oc*_) of up to 52 V and short-circuit current (I*sc*) density of 2.45 mA/m^2^ from water-TENG contact electrification^19^. Niu et al. developed the theory and the working mechanism of a sliding-mode TENG for both energy scavenging and self-powered sensor applications^20^. In another work, water drop energy was harvested by a sequential contact-electrification and electrostatic-induction process, and the output current and power density were 1.5 µA/cm^2^ and 20 mW/cm^2^ from tap water, respectively^21^. Tang et al. reported a mercury-based high-performance TENG with immediate energy-conversion efficiency using Kapton, copper electrodes, and mercury as the friction materials^18^. Recently, Zhao et al. were developed an ultrahigh electricity generation from low-frequency mechanical energy by efficient energy management and they produced over 7.5 kV energy through TENGs^22^. Besides, Yike et al. have been reported independent TENG results based on air-break down model charge excitation triboelectric nanogenerators and received the charge density of 2.38 m C/m^2^ using 4 µm thickness PEI film^23^. However, the focus of previous studies has been the enhancement of energy conversion rather than eco-friendliness and human health, especially regarding the central and peripheral nervous systems, which are serious considerations for environmental and living systems.

Zhu et al. developed a water-wave energy-harvesting system by an asymmetric screening of electrostatic charges on a nanostructured hydrophobic thin-film surface. The main focus was the liquid–solid electrification-enabled generator, which is based on a fluorinated ethylene-propylene thin film and free-standing-mode water, and a basic comparison with other modes was not performed^24^. Recently, our group reported sustainable green current generated by a fluid-based TENG (FluTENG) and compared contact and sliding modes using PTFE as an electrode. The triboelectric-power density values were 2.15 mW/m^2^ and 0.8 mW/m^2^ for the CS-FluTENG and LS-FluTENG, respectively^21,24^. Very recently, Yang et al. reported a water tank TENG fabricated with super-hydrophobic PTFE for efficient harvesting of water-wave energy using tap water, DI water, and NaCl solutions in reservoirs. The triboelectric open-circuit voltage (V_*oc*_) and short-circuit current (I*sc*) of tap water were 39.2 V and 2.25 mA/m^2^ due to the presence of mineral impurities in tap water. Although they achieved high V_*oc*_ and I*sc* from tap water, the polypropylene (PP) tank was very big, and the process is tedious^25^. The development of evaluation techniques is still needed for the accomplishment of higher TENG efficiency.

Higher hydrophobic rough surfaces are performed a pivotal role to generate instantaneous voltage, and currents when they interact with the water stream and splitting of water through a fast slip down process^26^. Similarly, in the present research work, we developed a simple, economically viable, and eco-friendly higher hydrophobic surface based on a PTFE membrane for a flexible tubing nanogenerator. The PTFE membrane was inserted inside of the flexible tube along with a copper electrode, and connected with a wire. The PTFE surface can work as a fluid TENG when Tap water (TW), and DI water have interacted abruptly and as continuous flow circulation. For simple acronyms and convenience, we named TW-Sh-PTFE-TENG as TW-Sh-TENG, TW-N-PTFE-TENG as TW-N-TENG, DI-Sh-PTFE-TENG as DI-Sh-TENG, and DI-N-PTFE-TENG as DI-N-TENG. The TW and DIw were used for the TW-N-TENG, and DIw-Sh-TENG, which are negatively charged according to the triboelectric series and are easily accessible as non-toxic and non-corrosive. TW-Sh-TENG was selected for the solid support due to its hydrophobicity, high negativity in the triboelectric series including chemical and thermal stability^27–30^.

## Results

### Structure design, and fabrication

The N-/Sh-TENG were fabricated by adhering the N-PTFE/Sh-PTFE films on the aluminum (Al) surface and then fixing it inside a flexible silicone tube (0.5 cm × 15 cm), which was named the N-/Sh-TENG cell. When a stream of Tap Water (TW) or DI water (DIw) was circulated through the N-/Sh-TENG inside of silicone tubing, the protected N-/Sh-TENG cell was received negative ions (electrons) from the TW or DIw streams and generate power due to the presence of N-PTFE/Sh-PTFE film. Subsequently, the water droplets were transformed into a positively charged electric double layer during circulation of the water stream^31^.

**Figure 1:**
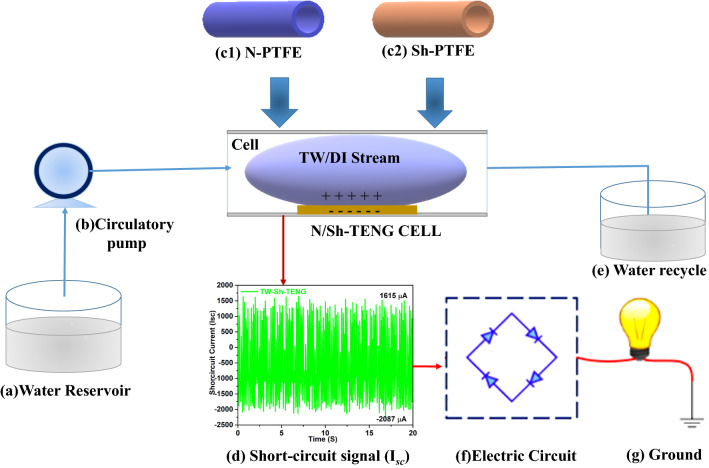
Schematic representation of the Sh-TENG, and N-TENG apparatus.

The water circulation system includes a long silicone pipe that was connected to a water tank as a reservoir (a), a circulation pump with a variable speed drive (b), and PTFE-TENG cell. As soon as the TW droplets were touched the PTFE membrane, a charge imbalance ascends between them. Consequently, when the droplet completely departures the cell, the electrons were transferred from the ground to the Al electrode through an external load, which generates electricity (d). The rectifier circuit (f) is used as an electrical coupling between the PTFE-TENG, and LED (g), as shown in Fig. 1.

### Fabrication of PTFE-TENG, and contact angle (CA) studies

**Figure 2:**
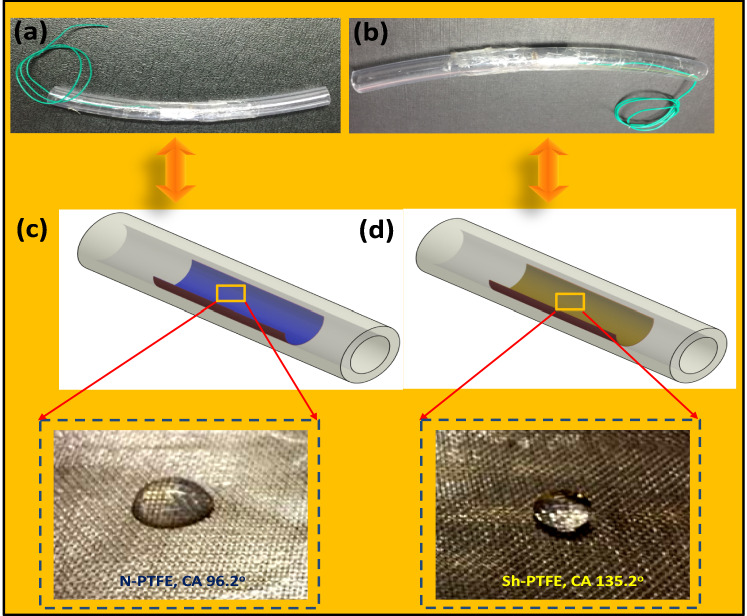
Digital photos of large droplets (~ 5 μl) placed on (**a**) N-TENG cell, (**b**) Sh-TENG cell, (**c**) N-PTFE (CA = 96°), (**d**) Sh-PTFE (CA = 135.2°).

A lotus-leaf-like Sh-PTFE film was prepared from N-PTFE film by spraying an acrylic thinner to create the super-hydrophobicity. The acrylic thinner was sprayed on N-PTFE and dried, this process was repeated to generate higher roughness through layer-by-layer deposition to generate super-hydrophobic N-PTFE film such as Sh-PTFE^32^. To determine the wetting performance of the as-prepared Sh-PTFE film, the water contact angle (CA), and water adhesion properties were studied, as shown in Fig. 2. The as-prepared Sh-PTFE showed excellent water repellency (CA = 135°) over the N-PTFE (CA = 96°, Fig. 2a, c). The water droplet (∼0.48 μl) is spherical on the modified Sh-PTFE surface (Fig. 2b, d) due to the generation of higher surface roughness like lotus leaf^33,34^.

### FE-SEM, AFM of N-PTFE, and Sh-PTFE

**Figure 3:**
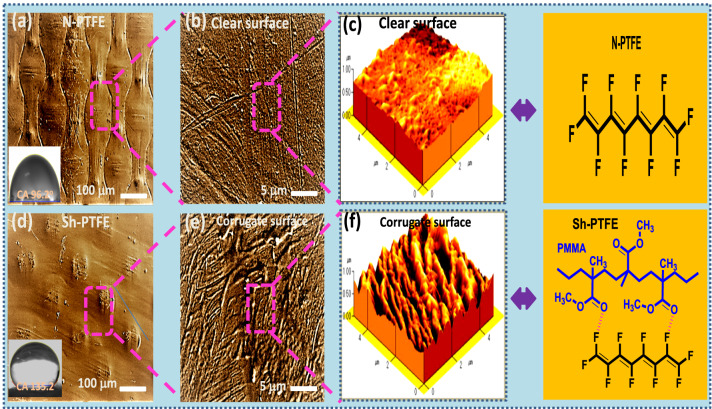
The SEM images showed (**a**, **b**) clean surface of N-PTFE(inset: CA of 96.2°), and (**d**, **e**) corrugated surface of Sh-PTFE, (inset: CA of 135.2°). The AFM images revealed (**c**) clear surface morphology on N-PTFE film (the height of the layer ~ 45 nm) (**f**) and corrugate morphology on Sh-PTFE film (the height of the layer ~ 113 nm).

The surface morphology of N-PTFE, and Sh-PTFE was determined by FE-SEM and AFM analyses, as shown in Fig. 3. A clean surface was found on the N-PTFE (Fig. 3a, b) and whereas on the Sh-PTFE showed micro-corrugated structures (Fig. 3d, e) due to the deposition of acrylic thinner by LBL depositions. Representative micro-corrugate channels were consistently spread on the N-PTFE surface and generated the super-hydrophobic Sh-PTFE^26^ which is the key functional material for generation power when interact with flow water. Besides, Fig. 3c, f were showed the Atomic Force Microscopy (AFM) images of N-PTFE(~ 45 nm), and Sh-PTFE(~ 113 nm) and which have been revealed the surface morphologies, and topographical orientations that can represent the L-B-L deposition of acrylic PMMA thinner on the Sh-PTFE surface. The higher surface roughness of Sh-PTFE was displayed superior V_*oc*_ and I_*sc*_ than N-PTFE because of the fast sliding nature when interacted with the water on Sh-PTFE. The FT-IR, XRD, and EDX analyses of N-PTFE and Sh-PTFE are discussed in the supporting information^27^.

### FT-IR, XRD, contact angle, and EDX (Energy dispersive X-ray spectroscopy) of N-PTFE, and Sh-PTFE films

#### FT-IR

**Figure 4:**
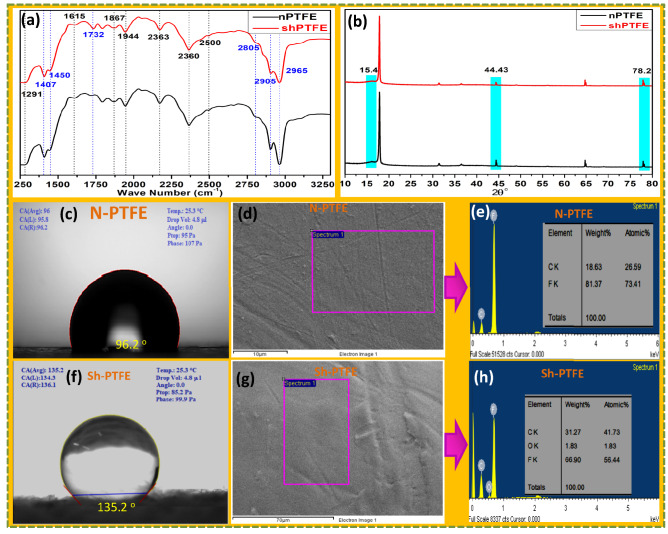
(**a**) FT-IR, (**b**) XRD, (**c**, **f**) CAs, and, (**d**, **g**) SEM-EDX, (**e**, **h**) elemental analysis of both N-PTFE and Sh-PTFE surfaces.

The FT-IR analysis showed the evidence of the modification of the Sh-PTFE from N-PTFE, as shown in Fig. 4a. Sh-PTFE showed new absorption peaks at 1407 cm^−1^, 1450 cm^−1^, 1732 cm^−1^, 2805 cm^−1^, 2905 cm^−1^, and 2965 cm^−1^ belong to C–(O)–F, CF, CH_2_, C=C, and –OH functional groups, respectively, due to formation of carboxylic acid on the Sh-PTFE surface after interaction of TW^23,24^. These peaks were strongly suggested that the N-PTFE was carbonized by the reaction of acrylic PMMA thinner, and made the Sh-PTFE as a super-hydrophobic rough surface.

#### XRD

The XRD results were obtained from the diffraction pattern, as shown in Fig. 4b. The N-PTFE exhibited typical crystalline peaks at 15.4°, 18.4°, 31.5°, 36.5°, 44.4°, and 78.2°, which are in agreement with the literature model^24^. The peak intensities for the Sh-PTFE were distorted due to the deposition of acrylic polymer thinner and changed the crystalline pattern. Except for these signals, the N-PTFE and Sh-PTFE patterns look similar and indicate the absence of any obvious crystallinity changes after the interaction with acrylic thinner^35,36^.

#### Contact angles (CA)

In order to prepare the substrates for surface modification using commercial PMMA acrylic thinner (Rust-Oleum).The PMMA hydrophobic thinner was deposited by spray coating and drying process and repeated to several time to achieved the desired thickness with layered nano pattern. For that, we used N-PTFE (CA 90.2°), for the deposition surface, as shown in Fig. 4c. After deposition, the surface was exhibited a dramatic increase in CA by sessile drop method, Sh-PTFE exhibiting super hydrophobicity, having CA 135.2° with water droplet, as shown in Fig. 4f. The detailed fabrication process was depicted in Supporting Information, SI-2.

#### EDX

Energy-dispersive X-ray (EDX) spectroscopy analyses revealed that super hydrophobicity that was achieved by the chemical deposition of acrylic thinner on the Sh-PTFE, as shown in Fig. 4g from N-PTFE Fig. 4d. The atomic %, and weight % of F intensity was decreased from 81.37% (Fig. 4e) to 66.9% (Fig. 4h).with acrylic thinner deposition by chemical reaction, whereas the atomic %, and weight % of C, and O increased from 18.63 to 31.27%, and 0 to 1.83%, respectively. The significant changes in the surface morphologies were achieved by acrylic thinner due to the increase in the oxygen-containing functional groups such as –OH, and –COOCH_3_ due to the interaction of CF_2_ groups of N-PTFE, Fig. 4e. The SEM images of N-PTFE, and Sh-PTFE were suggested the super-hydrophobicity^37–40^. Significant morphological changes were observed on the Sh-PTFE rather than the N-PTFE surface, as shown in Fig. 4h, which indicated that oxygen atoms were existing on the Sh-PTFE surface.

### Discussion

#### Mechanism of the chemical-reaction pathway between the water N-TENG and Sh-TENG, and their FT-IR, and XRD analyses

##### Mechanism

**Figure 5:**
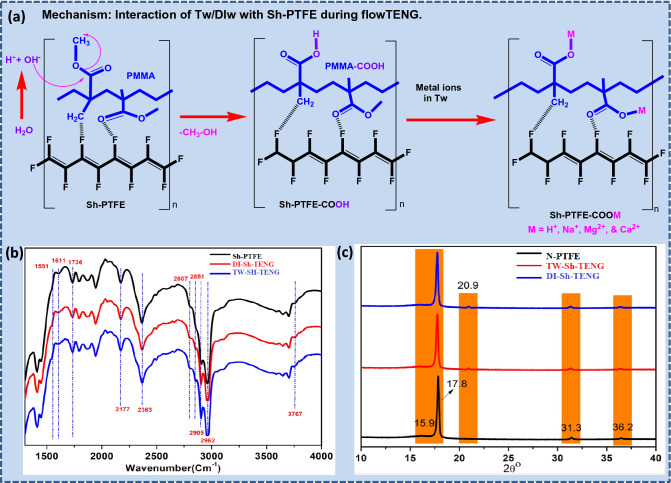
Schematic representation of mechanism of (**a**) Sh-PTFE with water molecules and nucleophilic elimination of CH_3_OH; (**b**) FT-IR; and (**c**) XRD spectra of N-PTFE and Sh-PTFE.

The output voltage, and currents of flow TENG were generated from the strong interaction of TW water with Sh-TENG during TW water circulation, where the water can split into hydronium (H^+^), and hydroxide ions (OH^−^). To observe the dynamic interactions between CF_2_ of Sh-PTEFE and water at atomic level interactions were noted, as shown in Fig. 5a^41^. In particular, the OH– ions were replaced methoxy ions (–OCH_3_) by the nucleophilic substitution and elimination mechanism at the PMMA spacer, and made into terminal carboxylic acids. Subsequently, the dissolved metal ions in TW were temporarily chelated with -COOH groups, and produced metal salts –COOM (i.e. M = H^+^, Na^+^, Mg^2+^, and Ca^2+^). Next, the quantum chemical molecular dynamics (QCMD) were investigated by Onodera et al. to identify the formation of carbonyl functional groups on the back bone chain of PTFE, and water was the counter fluid during the tribochemical reaction^34^. Besides, Pletincx et al. have reported the -COOH groups have obtained after the interaction of water molecules with a PMMA surface, and elimination of CH_3_OH as a by-product. Thus, the FT-IR analysis of our Sh-PTFE is well judged with their reported information^42^.

**Figure 6:**
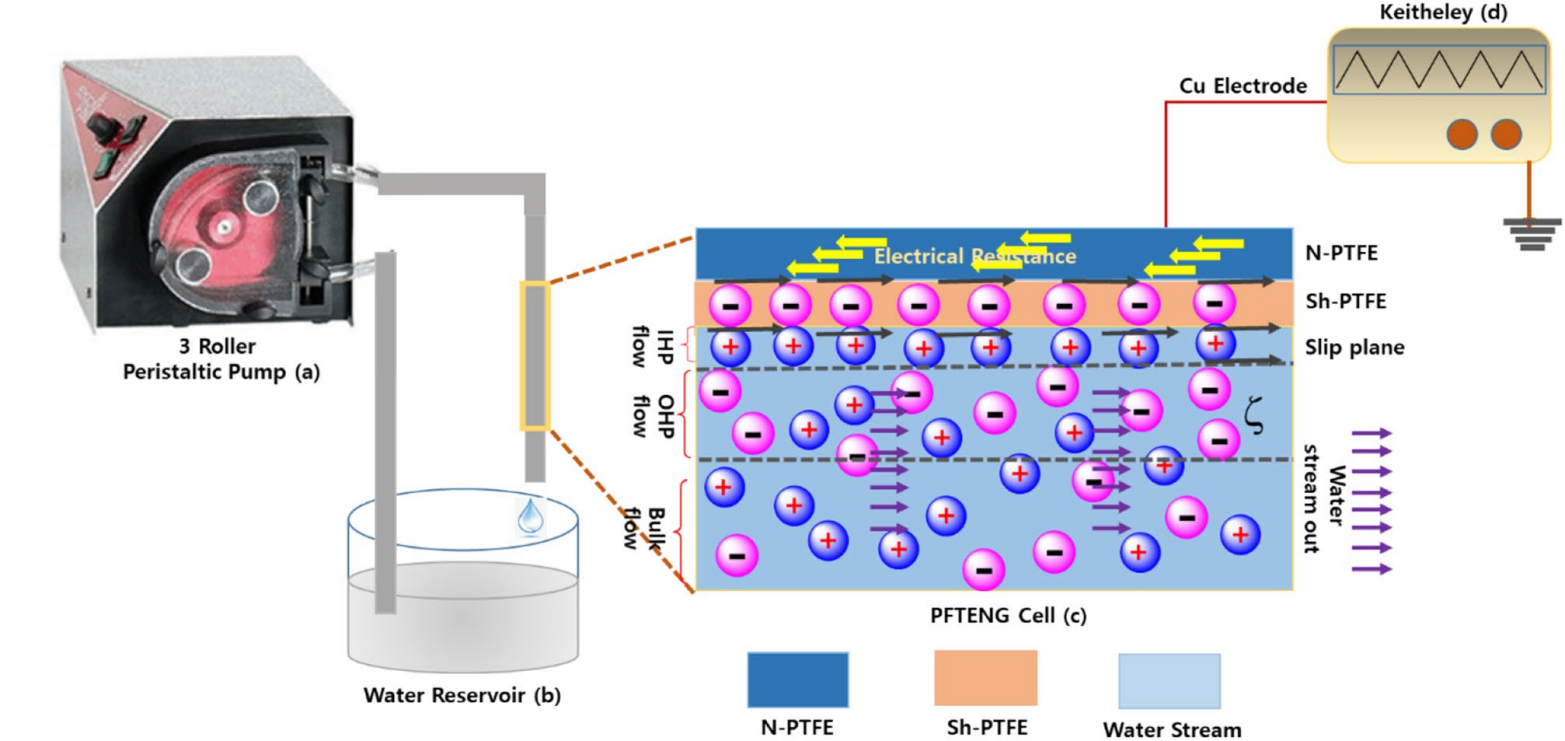
Mechanistic approach to generate the Electric double layer (EDL) for power supply through (**a**) peristaltic pump, (**b**) water reservoir (**c**) TENG cell showing the EDL = IHP + OHP at the interface of solid–liquid and the reduction of the zeta potential after the IHP to the bulk segment of the flow liquid. (**d**) Electrometer. (Supporting information showed the picture of live experimental set-up of for the proposed flow TENG Apparatus).

Figure 5b shows the FT-IR spectra of N-PTFE, TW-Sh-TENG, and DI-Sh-TENG were revealed splitting and generation of H^+^ ions on the Sh-PTFE surface. The generated H^+^ ions are responsible to produce V_*oc*_ and I_*sc*_ through a charge transfer mechanism during the interaction of TW with the Sh-PTFE surface, as shown in Fig. 6a. From the N-PTFE, two strong bands occurred near 1157 cm^−1^ and 1218 cm^−1^, which correspond to the main –CF_2_ chains of the symmetric and anti-symmetric stretching bands of v(C–C) vibration manifests in the form of modulation at 1258 cm^−1^^43–45^.

After the interaction of TW with the Sh-PTFE surface, the intensity of anti-symmetric stretching bands of the CF_2_ was reduced due to the interactions between the H^+^ ions in water. Consequently, the new stretching bands have appeared at 1551 cm^−1^, 1611 cm^−1^, 1736 cm^−1^, 2177 cm^−1^, 2363 cm^−1^, 2807 cm^−1^, 2851 cm^−1^, 2962 cm^−1^, and 3376 cm^−1^, which were corresponding to PMMA-COOH, and –OH functional groups owing to the formation of C^+^–F^−^–H^+^ transition bonds, respectively. In the case of PTFE-COOH, a medium band appeared at 1551 cm^−1^, which belongs to C=C of PTFE, which was interacted with water, and form a –C–(OH)–C– bond. A band appeared at 1736 cm^−1^ of Sh-TENG because of the absorption by the –CO– of COOH. Besides, a strong broad absorption band appears at 3309 cm^−1^, which represents the presence of –CF=C–COOH, and revealed the formation of PMMA-COOH after hydrolysis of PMMA methyl ester, as explained in Mechanism-2^44^. As a result, the mechanistic approach is strongly suggested by the interaction of the FT-IR spectrum was strongly suggested a mechanistic approach for the Sh-TENG, and N-TENG interaction with TW, and DIw^25,41,45,46^.

After fluid TENG experiments, the dissolved salts in TW such as (M = H^+^, Na^+^, Mg^2+^, and Ca^2+^) and DI were deposited on the surface of N-PTFE and Sh-PTFE and quantified the degree of crystallinity by the XRD analysis, as shown in Fig. 5c. The N-PTFE exhibited typical XRD peaks at 15.9°, 17.8°, 31.3°, and 36.2°, which are in agreement with the literature^23^. The new peak was observed at 20.9°, and also, peak intensities were reduced from Tw-Sh-TENG to DI-Sh-TENGs due to the formation of -COOH groups on the Sh-PTFE surface^35^.

### Mechanical pathway for the characteristic discontinuous current generation

Figure 6 showed the performance of the flow TENG using various TW-Sh-TENG, TW-N-TENG, DI-Sh-TENG, and DI-N-TENGs. To generate the pulsatile stream, a three-roller peristaltic drive (Ecoline VC-360) was used in this research, Fig. 6a. The long go through silicon tubing with the N-PTFE/Sh-PTFE-TENG cells as described in the material section and which were used for the fluid passage which is constantly flowing the working fluid, Fig. 6b. To quantify the charge generation mechanism in the N-PTFE/Sh-PTFE-TENG cells from the fluid stream, which is compared with the Chapman-Stern layer mathematical model that was combined the hydrodynamics and electrostatic stimulation in the PTFE-TENG solid–liquid interface was elucidated with the aid of electrical double layer formation^47^. When the flowing liquid starts rolling on the silicon tubing, the Si–O–H were momentarily protonated and accommodates the opposite counter ions of the flowing liquid at the solid–liquid boundary. Owing to the rigid (no-slip, U = 0) border ailment at the barrier, the extremely intense ions were immobile within the solid–liquid interface through the working fluid flows, Fig. 6c. The hydrophobic properties of the N-PTFE/Sh-PTFE surfaces provide slip condition (U ≠ 0) at the solid–liquid interface facilitates extremely focused ions to be moveable in the range of slip-up plane, and hard border and then enhances the liquid flow velocity^48,49^. The ions were at the slip plane that maintains remain the similar velocity along with liquid flow above the N-PTFE/Sh-PTFE surfaces. After the interaction of the flow working fluids, the generated V*oc* and I*sc* were quantified using Techtronics digital oscilloscope, Fig. 6d (Supplementary information 1 and 2).

### Results of N-TENG, and Sh-TENG through a 3 roller peristaltic circulation pump

The liquid used for the flow TENGs is water-based and is positively charged according to the triboelectric series. The liquid is easily accessible, non-toxic, and non-corrosive. N-PTFE was chosen on the solid side owing to its hydrophobicity with high negativity in the triboelectric series, and chemical constancy^26^. To develop N-TENG (Fig. 2a), and Sh-TENG (Fig. 2b), we placed normal PTFE (N-PTFE) (Fig. 2c), or super-hydrophobic PTFE (Sh-PTFE) (Fig. 2d) films with a thickness of 0.08 mm on general-purpose aluminum tape. The flow TENG was fabricated by adhering the N-PTFE/Sh-PTFE layer to an aluminum (Al) surface and then fixed inside a general-use flexible silicone tube (0.5 cm × 15 cm). The Sh-PTFE was obtained by deposition of acrylic PMMA Previously, we described upon the contact the liquid with PVDF membrane and generated power output through peristaltic pump^50^, similarly, the present work of N-PTFE/Sh-PTFE surfaces, due to its CH_3_ groups were transformed into highly electronegative of CH_2_– so thus attracting the positive counter ions following the electrostatic induction and triboelectrification. This phenomenon motives the development of an Electric Double Layer (EDL) within the sliding plane. The EDL contains Inner Helmholtz Plane (IHP) where the positive counter ions can move peripherally along with the flow direction over the N-PTFE/Sh-PTFE surfaces, and the other one Outer Helmholtz Plane (OHP) whereas the mobile positive ions and the counter ions can transpire together, as shown in Fig. 6. In the IHP, the drive of the counter ions was strongly influenced by the common hydrodynamic liquid stream in the pipe flow. The counter ions can move superficially alongside the slide range of the boundary partition but showed uncertainty to be gathered in the OHP or in the bulk liquid. In the case of OHP, the reverse polarized could travel to the bulk liquid. For instance, the flowing liquid was viscid, and incompressible by the Newtonian liquid, then peristaltic flow at the PF-TENG cell-liquid interface, the continuity and Navier Stokes equations clench worthy and can be modified for the ion's drive in the IHP^51,52^.

1 $$\frac{\partial \rho }{{\partial t}} = \nabla \cdot (\rho v_{fluid} ) = 0$$

where ρ and υ_fluid_ remain the density, and velocity of the working fluid, correspondingly.

The modified Navier–Stokes equation

2 $$\rho \frac{{\partial v_{fluid} }}{\partial t} + \rho (v_{fluid} \cdot \nabla )v_{fluid} = \nabla \cdot [ - \rho I + \mu (\nabla v_{fluid} + (\nabla v_{fluid} )^{T} )] + F_{n}$$

where *F*_*n*_ was the body force vector forced on the liquid. In our system, the body force was the applied electrostatic magnetism power in usual direction between the surface electrons of the PTFE surface, and the positive charges in the IHP. Allowing to the Guoy–Chapman theory^53^, this magnetism power could be in the rolling form

3 $$F_{n} = \frac{{(qE_{n} = A_{p} \sigma^{2} )}}{{(\varepsilon_{0} \varepsilon_{1} )}}$$

Here, q is the electrical charge, A_p_ is the effective contact area, *σ* is the surface charge density, ε_0_ and ε_1_ are the dielectric permittivity of the vacuum, and working liquid. Where at the PTFE liquid interface, the liquid was positively charged owing to high dielectric constant rendering to Cohen's rule^54^.

4 $$\rho = 1.5 \times 10^{ - 5} (\varepsilon_{1} - \varepsilon_{2} )$$

Here, ε_2_, ρ are the dielectric constant of the Sh-PTFE, and the space charge density, correspondingly. The Debye characteristic length at the IHP was expressed by,

5 $$\lambda_{D} = \frac{{\xi \varepsilon_{0} \varepsilon_{1} }}{\sigma }$$

where ξ was the Zeta potential as represented in Fig. 6c, it is gradually decreased from IHP to working liquid. The shear stress τw of the liquid was enacted on the wall can be written by Ref.^55^.

6 $$\tau_{w} = \frac{{2\mu v_{i} }}{{R = \lambda_{D} }}$$

In Eq. (6), the υi was the ion velocity that is equal to the υ fluid at the IHP, and the circle of the flow tube R was substituted by the Debye Length. So, the implicit shear force, and the derived ion velocity was expressed by Eqs. (7) and (8), correspondingly.

7 $$F_{s} = A_{p} \tau_{w} = A_{p} \mu \frac{{2v_{i} }}{{\lambda_{D} }}$$

8 $$v_{i} = \frac{{\tau_{w} \lambda_{D} }}{2\mu }$$

Due to the slide boundary condition when applied on the over the PTFE surface the ions velocity υi was multiplied by the factor of (1 + bλD)^56^, where b was the slip length. So, the ion velocity becomes

9 $$v_{i} = \frac{{\tau_{w} \lambda_{D}^{2} }}{{2\mu (\lambda_{D} + b)}}$$

The static rubbing force in the solid PTFE surface that was reacted oppositely to the shear force was equal to the *F*_*n*_, and multiplied with the friction coefficient *μ*_*s*_ subsequently followed by the Amonton's law of stationary resistance

10 $$F_{f} = \mu_{s} F_{n}$$

The ions were started to slip against the solid border of the film once Fs > Ff. Alternatively, on the solid surface the electrons were pulled by the sheer force of the ions, but the velocity was delayed due to the electrical resistance of the solid. During this time, the momenta was preserved for both the counter ions, and electrons at the interface. The electrons were pulled back by the ions affecting comparative distance thus velocity difference was induced the potential difference in the interface, and short circuit current by the external load. The Drude model is adopted by the solid-state physics showed electrons on the solid surface

11 $$\frac{{d(mv_{e} )}}{dt} = \frac{{ - mv_{e} }}{{\tau + qE_{ext} }}$$

where υ_*e*_, τ, _*q*_*E*_*ext*_ were velocity of electrons, Drude relaxation time, and applied outside arena. In this model, the electrostatic attraction was strong enough that the electrons were dragged by the quicker ions in the IHP. As the external applied was zero, so Eq. (10) reduces to

12 $$\frac{{d(mv_{e} )}}{dt} = \frac{{ - mv_{e} }}{\tau }$$

The momenta are preserved by the electrons across the interface,

13 $$\frac{{F_{s} + d(mv_{e} )}}{dt} = 0$$

Combining both Eqs. of (10), and (11) the electron velocity becomes as follows

14 $$v_{e} = \frac{{F_{s} \tau }}{m}$$

Now, the net amount of induced charges in the Sh-PTFE surface owing the electrostatic potential triggered by the comparative distance between the electrons, and flow ions,

15 $$Q_{s} = \sigma_{0} S = \sigma_{0} wx$$

The prompted net electric power in the solid surface is provided by

16 $$I_{s} = \frac{dQ}{{dt}} = \sigma_{0} w\frac{dx}{{dt}} = \sigma_{0} w\Delta v = \sigma_{0} \pi a(v_{i} - v_{e} )$$

The resultant PTFE surface electric potential owing to the electrical resistance, R, and electric power is

17 $$\psi_{s} = I_{s} R = \frac{{I_{s} }}{{bK_{s} }}$$

where b is the distance of the _q_E_*ext*_ and K_s_ is the surface conductivity.

Based on the resulting Eqs. (9), (14), and (17) for the ions velocity, electrons velocity, and surface potential of the PTFE surfaces, correspondingly, the triboelectrification owing to the pulsatile flow over the PTFE surfaces can be explained.

### Results of flow-TENG

**Figure 7:**
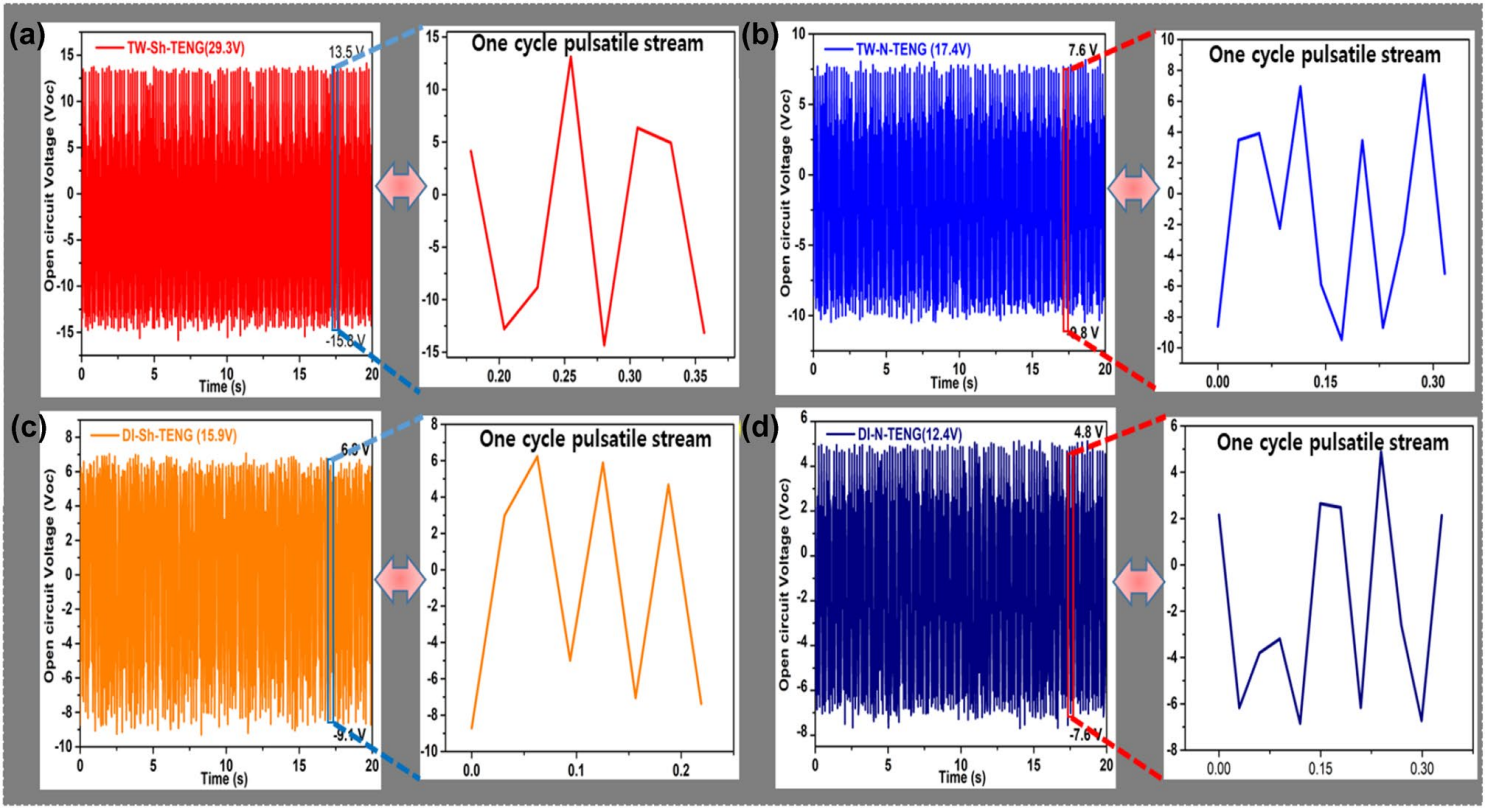
The open-circuit voltages (V_*oc*_) of (**a**) TW-Sh-TENG; (**b**) TW-N-TENG; (**c**) DI-Sh-TENG; (**d**) DI-N-TENG and their single to three pulsatile open-circuit voltages (V_*oc*_) showed in the inset images.

**Figure 8:**
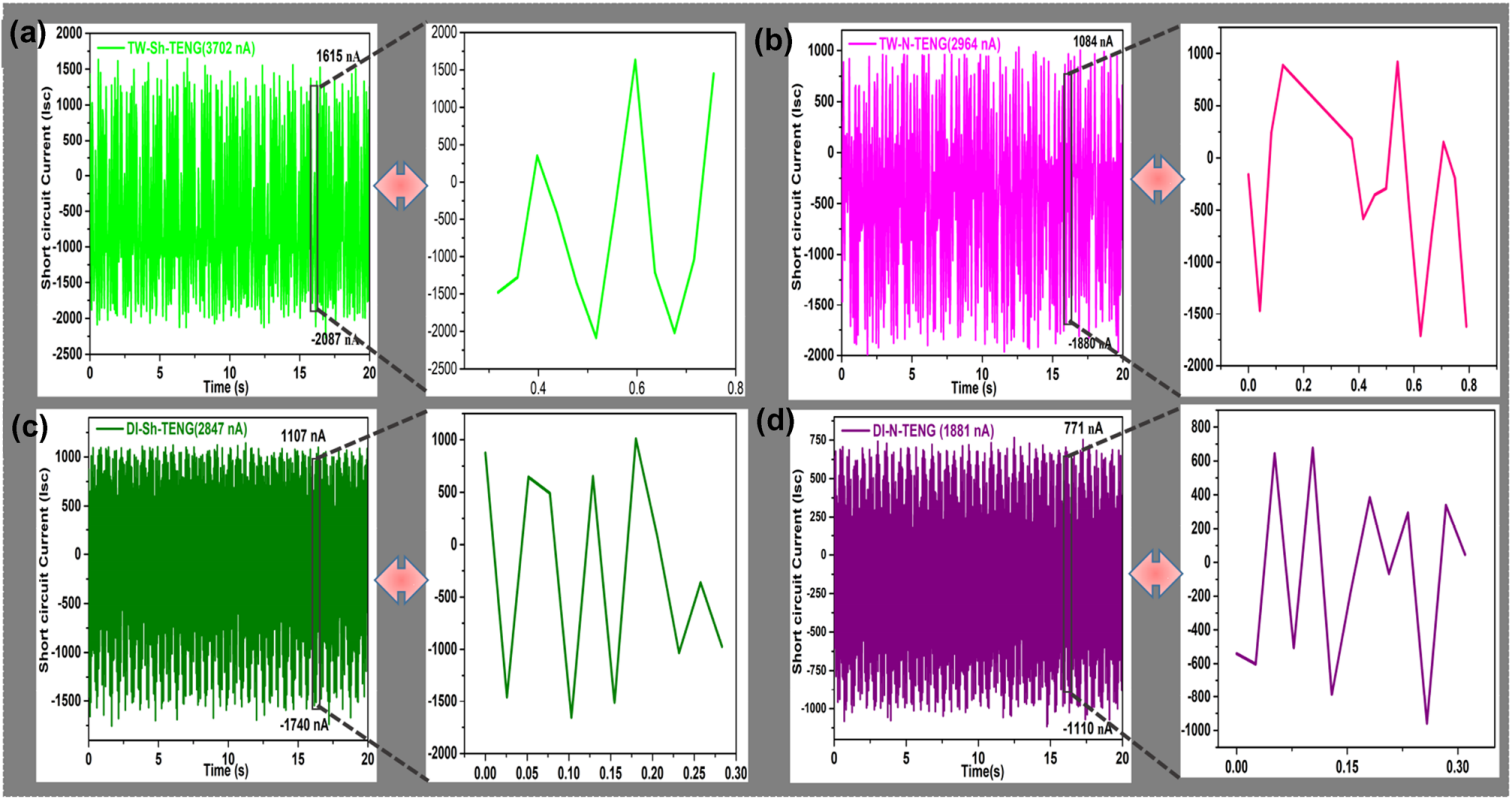
The short-circuit current (I_*sc*_) of (**a**) TW-Sh-TENG; (**b**) TW-N-TENG; (**c**) DI-Sh-TENG; (**d**) DI-N-TENG and their single to three pulsatile open-circuit voltages (V_*oc*_) showed in the inset images.

Also, the contact electrification in metal-dielectric cases was strongly studied by Z. L. Wang et al. where they have elaborated various metal-dielectric cased to generate the power output through a contact-electrification process^57^. The present studies revealed that the electrical outputs of the proposed Flow-TENG surfaces are demonstrated in this section. All measurements refer to an operating speed of 350 rpm/min, which can be manually controlled by the tubing pump (Ecoline VC-360). The Flow-TENG was produced instantaneous peak-to-peak values of open-circuit voltages (Voc), and short-circuit currents (Isc) and compared with different unmodified and modified surfaces such as N-PTFE and Sh-PTFE, respectively. For simple acronyms and convenience, we named TW-Sh-PTFE-TENG as TW-Sh-TENG, TW-N- PTFE-TENG as TW-N-TENG, DI-Sh-PTFE-TENG as DI-Sh-TENG, and DI-N-PTFE-TENG as DI-N-TENG. It is observed that the V*oc* and I*sc* of Flow-TENGs, TW-Sh-TENG, TW-N-TENG, DI-Sh-TENG, and DI-N-TENG were produced of 29.3 V (Fig. 7a), 17.4 V (Fig. 7b), 15.9 V (Fig. 7c), and 12.4 V, (Fig. 7d). Next, the I*sc’s* were obtained 3.7 µA (Fig. 8a), 2.9 µA (Fig. 8b), 2.7 µA (Fig. 8c), and 1.8 µA (Fig. 8d), from TW-Sh-TENG, TW-N-TENG, DI-Sh-TENG, and DI-N-TENG, respectively. The attributed values were suggested that the V*oc* of TW-Sh-TENG was showed 139% higher than that of V*oc* of DI-Sh-TENG, and the I*sc* of TW-Sh-TENG is 101% than DI-N-TENG, respectively, due to the lack of dissolved ions in DI water, also, sufficient roughness, and super-hydrophobicity on the N-PTFE surface. The constant Voc and Isc were observed in all pulsatile streams for one to five cycles which are represented in inset images, as shown in Figs. 7 and 8. Besides, the mechanistic explanation was strongly suggested the TW-Sh-TENG was shown superior V*oc, *and I*sc* over DI-Sh-TENG because of higher dissolved mineral salts in TW that can further enhance the generation of higher power output compared to DIw-TENG^57–59^.

### Power output studies

**Figure 9:**
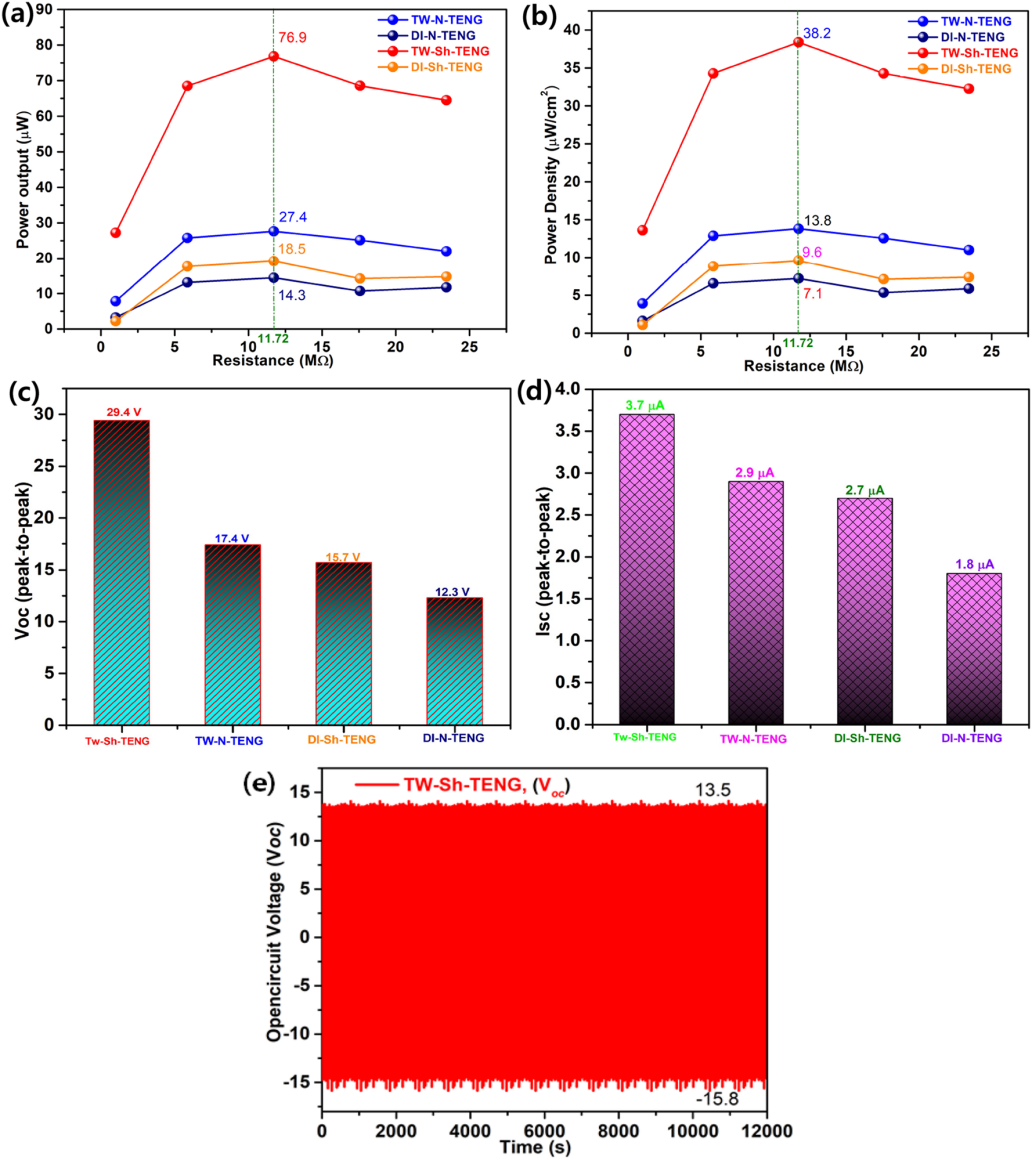
The power output (**a**); power density (**b**) and comparison of overall V_*oc*_ (**c**) and I_*sc*_ (**d**) of TW-Sh-TENG, TW-N-PTFE-TENG, DI-Sh-TENG and DI-N-TENG; (**e**) stability and durability test for 12,000 s were also conducted to confirm the durability and sustainability.

Subsequently, the performance under different external loads was investigated. The maximum power output was achieved by TW-Sh-TENG using TW using various load resistances of 1 MΩ to 23.44 MΩ. The dependence of the power output curves and power density as functions of the resistance are shown in Fig. 9. When increasing the load resistance, the power output increased up to a maximum value and then decreased. The power reached its maximum value at an external load resistance of 11.72 MΩ (green vertical lines). The maximum powers were obtained at 11.72 MΩ for 76.9 μW (TW-Sh-TENG), 27.4 μW (TW-N-TENG), 18.5 μW (DI-Sh-TENG), and 14.3 μW (DI-N-TENG, respectively, as shown in Fig. 9a. On the other hand, the maximum power densities were obtained at 11.69 MΩ for 38.2 μW/cm^2^ (TW-Sh-TENG), 13.8 μW/cm^2^ (TW-N-TENG), 9.6 μW/cm^2^ (DI-Sh-TENG), and 7.1 μW/cm^2^ (DI-N-TENG), respectively, as shown in Fig. 9b^57–59^. The TW-Sh-TENG produced significantly higher power output over normal TW-N-TENG (178%), which verified the importance of super-hydrophobicity in water-based TENGs^60,61^.

Next, the peak-to-peak open-circuit voltages were 29.4 V for TW-Sh-TENG, 17.4 V for TW-Sh-TENG, 15.7 V for DI-Sh-TENG, and 12.3 V for DI-N-TENG, respectively, as shown in Fig. 9c. The short-circuit currents were 3.7 μA for TW-Sh-TENG, 2.9 μA for TW-Sh-TENG, 17.4 V for TW-Sh-TENG, 2.7 μA for DI-Sh-TENG, and 1.8 μA for DI-N-TENG, respectively, as shown in Fig. 9d. The Voc and Isc have suffered a predictable drop in magnitude from TW-Sh-TENG to DI-N-TENGs, as shown in Fig. 9c, d. The output power of DI-N-TENG dramatically decreased by 4 times compared to DI-Sh-TENG from 76.9 to 18.5 μW, and the power density decreased from 38.2 to 9.6 μW/cm^2^^62^. The power output of TW from the TW-Sh-TENG is up to 300% higher than that with DI-TENG. Similarly, TW-N-TENG produced a higher power output of 94% with TW compared to DI. In the case of power density, we observed higher values from TW-Sh-TENG than TW-N-TENG and the power densities were 178% and 33%, respectively due to the H^+^ ions expelled F- ions as HF from the surface^56,63^. More importantly, TW contains dissolved mineral salts that can enhance the power output compared to DI water. Besides, the performance stability of the designed TW-Sh-TENG has been tested for 12,000 cycles and received the constant voltage, as shown in Fig. 9e. The test results strongly supported our idea for using the TW-Sh-TENG has been produced superior Voc and Isc compared with other designated flow TENGs due to the higher irregular surface morphology, and super-hydrophobicity^64,65^.

## Methods

### Material descriptions

The liquid was used for the flow TENGs is water-based and is positively charged according to the triboelectric series. The liquid is easily accessible, non-toxic, and non-corrosive. N-PTFE was selected for the solid surface owing to its hydrophobicity, higher negativity in the triboelectric series, and chemical stability^26^. To develop N-TENG (Fig. 2a), and Sh-TENG (Fig. 2b), we placed normal PTFE (N-PTFE) (Fig. 2c), or super-hydrophobic PTFE (Sh-PTFE) (Fig. 2d) films with a thickness of 0.08 mm on general-purpose aluminum tape. The flow TENG was fabricated by adhering the N-PTFE/Sh-PTFE layer to an aluminum (Al) surface and then fixed inside a general-use flexible silicone tube (0.5 cm × 15 cm). The Sh-PTFE was obtained by deposition of acrylic PMMA thinner (Rust-Oleum Corporation, USA).

To characterize the surface properties, the contact angle (θ) was used. The contact angle of DI water on the Sh-PTFE surface was conducted with a DSA 100 Goniometer (error ± 1°) using the sessile-drop method (droplet volume: 0.48 μl) (KRUSSG mbh, Hamburg, Germany). The sample surface morphology was determined by a Hitachi cold FE-SEM microscope working at 10 kV. 2.2. The functional groups were measured by a Nicolet 6700 FT-IR, Diffraction data were acquired with a Rigaku high-power XRD. The images of the Multimode V AFM microscope (VEECO, US) were treated using a flattening algorithm in Nanoscope software, and the surface roughness average (Ra) was calculated using the instrument vendor’s software.

A simple electric circuit was designed to evaluate the load features, and harvest performance of the liquid nanogenerator through flow TENGs. The circuit that includes with a full bridge rectifier, a capacitor for energy storage to correct the cycled positive and negative signals, and store the transformed electricity. Tektronix TBS1102B Digital Oscilloscope was used to analyze the generated Voc and Isc^66–68^.

### Surface modification and characterization

The surface hydrophobicity of the solid tubing plays an important role in the output performance of Sh-TENG because the with-holding of liquid droplets, which could monitor the charges on the solid surface and thus reduce the power output. Since N-PTFE does not have high hydrophobicity, surface modification is necessary to enhance it. The Sh-PTFE was prepared by spraying acrylic thinner in mineral spirits and heated at 50 °C for 30 min (see the supporting information).

### Super hydrophobicity and contact angle (CA)

Hydrophobicity and super hydrophobicity behaviours of N-PTFE, and Sh-PTFE were examined via the CA measurement technique through a sessile drop of water. It was achieved that as received Sh-PTFE is exhibited super hydrophobic nature from N-PTFE due to increasing roughness on the surface by deposition of PMMA spacer. The PMMA spacer contains methyl ester groups which can enhance the surface tension to increase the contact angle. In particular, the CAs of N-PTFE and Sh-PTFE were 96° and 135.2°, respectively.

## Conclusion

For the first time, we introduced flexible silicone-pipe TENGs based on TW, and DI water. The fabrication of N-PTFE/Sh-PTFE films that were fixed inside the flexible silicone pipe has advantages that include easy handling, cheapness, eco-friendliness, and portability. The proposed Sh-PTFE-TENG is highly durable for circulation of tap water to generate higher V_*oc*_ and I_*sc*_. The Sh-PTFE surface was achieved by a very simple method of spray coating PMMA acrylic thinner. Water plays a pivotal role on the surface of N-PTFE, where nucleophilic substitution occurs at the terminal CF_2_ group to form a carboxylic acid group. At the Sh-PTFE surface, the water can hydrolyze the methoxy group, which leads to the formation of methanol and a carboxylic acid functional group. The interaction of water molecules is critical, which was elucidated through the mechanism and experiment and confirmed by FT-IR, and XRD analyses.

The TW-Sh-TENG, and TW-N-TENG were shown V_*oc*_*,* and I_*sc*_ of 29.4 V, and 3.7 µA and 17.4 V, and 2.9 µA, respectively. The V_*oc*_ and I_*sc*_ of DI-Sh-TENG and DI-N-TENG were achieved of 15.7 V, and 2.743 µA and 12.3 V, and 1.836 µA, respectively. The performance under different external loads was investigated, resulting in maximum power of 76.9 μW for TW-Sh-TENG, and 27.6 μW for TW-N-TENG, which correspond to power densities of 38.2 μW/cm^2^, and 13.8 μW/cm^2^ at 11.7 MΩ. The output power and power density dramatically decreased by 4 times from 76.9 to 18.9 μW, and from 38.3 to 9.6 μW/cm^2^ compare to the TW-Sh-TENG with DI-N-TENG. The TW-Sh-TENG produced significantly higher output power over the normal surface (178%), it indicated that it is strongly needed the development of super-hydrophobic surfaces for fast ionic interactions in water-based TENGs.

## Supplementary Information

Supplementary Information.
