# Supplementary material for: Harvesting of flow current through implanted hydrophobic PTFE surface within silicone-pipe as liquid nanogenerator
Source: Sci Rep. 2022 Mar 8;12:3700. doi: 10.1038/s41598-022-07614-5 (PMC8904805; doi:10.1038/s41598-022-07614-5)
Supplement: Supplementary file 1 — Supplementary Information. [file 41598_2022_7614_MOESM1_ESM.docx]

**Supporting Information**

**Live experimental set-up of for the** **Harvesting of Flow Current through Implanted Hydrophobic PTFE Surface within Silicone-Pipe as Liquid Nanogenerator**

**SI**. Live experimental set-up and mechanistic approach to generate the Electric Double Layer (EDL) for power supply through water stream through peristaltic circulatory pump.

SI.2. Fabrication process of N-PTFE to Sh-PTFE through (a)spray coating of PMMA Acrylic Thinner; (b) Sh-PTFE; (c) CA measurement by sessile dropping; (d) water drop on Sh-PTFE;(e) N-PTFE CA 96 ^o^; (f) Sh-PTFE CA 135.2^o^.
